# Mertk Deficiency Affects Macrophage Directional Migration via Disruption of Cytoskeletal Organization

**DOI:** 10.1371/journal.pone.0117787

**Published:** 2015-01-24

**Authors:** Yong Tang, Shen Wu, Qian Liu, Jiayi Xie, Jingxue Zhang, Dong Han, Qingxian Lu, Qingjun Lu

**Affiliations:** 1 Beijing Institute of Ophthalmology, Beijing Tong-Ren Hospital, Capital Medical University, Beijing 100069, China; 2 Beijing Tong-Ren Eye Center, Beijing Tong-Ren Hospital, Capital Medical University, Beijing 100069, China; 3 Beijing Ophthalmology and Visual Science Key Laboratory. Beijing 100069, China; 4 National Center for Nanoscience and Technology, Beijing 100190, China; 5 Department of Ophthalmology and Visual Sciences, University of Louisville School of Medicine, 301 E. Muhammad Ali Blvd, Louisville, KY 40202, United States of America; Vanderbilt University Medical Center, UNITED STATES

## Abstract

Mertk belongs to the Tyro3, Axl and Mertk (TAM) family of receptor tyrosine kinases, and plays a pivotal role in regulation of cytoskeletal rearrangement during phagocytosis. Phagocytosis by either professional or non-professional phagocytes is impaired in the Mertk deficient individual. In the present study, we further investigated the effects of Mertk mutation on peritoneal macrophage morphology, attachment, spreading and movement. Mertk-mutated macrophages exhibited decreased attachment, weak spreading, loss of spindle-like body shape and lack of clear leading and trailing edges within the first few hours of culture, as observed by environmental scanning electron microscopy. Time-lapse video photography recording showed that macrophage without Mertk conducted mainly random movement with oscillating swing around the cell body, and lost the directional migration action seen on the WT cells. Western blotting showed a decreased phosphorylation of focal adhesion kinase (FAK). Immunocytochemistry revealed that actin filaments and dynamic protein myosin II failed to concentrate in the leading edge of migrating cells. Microtubules were localized mainly in one side of mutant cell body, with no clear MTOC and associated radially-distributed microtubule bundles, which were clearly evident in the WT cells. Our results suggest that Mertk deficiency affects not only phagocytosis but also cell shape and migration, likely through a common regulatory mechanism on cytoskeletons.

## Introduction

Cell migration is a highly orchestrated process throughout the embryonic development, and during adult wound healing, tissue repair and regeneration, immune responses, and tumor metastasis [1]. Macrophage is a highly mobile cell type and can migrate in any given direction in response to specific signals, during which the shape of cell body is changed and a driving force within the moving cells is generated, largely through cytoskeletal rearrangement and dynamic protein reaction. In general, mammalian cell migration is considered as a continuous process of cell polarization, protrusion, adhesion formation at the leading edge and retraction at the trailing edge.

Cytoskeletons consist mainly of filamentous actin and microtubules. At the leading edge, monomer actins rapidly polymerize to form filamentous bundles that drive the membrane protrusion on a migrating cell [2–4]. There are two major types of membrane protrusion, i.e., lamellipodia and filopodia. Both forms are driven and supported by actin filaments, while the long parallel bundles form figure-like filopodia, the branching filamentous networks shape the flatten form of lamellipodia [5]. Formation of actin filements in lamellipodia and filopodia is regulated through a serial signaling cascade and the Rho family of small guanosine triphosphate (GTP)-binding proteins (GTPases), such as Rac, and Cdc42, is critical for protrusion formation of lamellipodia and filopodia. Both can activate downstream WAVE/WASP proteins that further stimulate the Arp2/3 complex to induce actin polymerization [1,5]. Another key signaling pathway that regulates cell migration, especially toward chemoatractant gradients, involves the PI3Ks and Pten phosphatase, which collaboratively regulate the local contents of phosphoinositides PI(3,4,5,)P_3_ and PI(3,4)P_2_. Both are required for localizing the Rac and Cdc42 activity on the leading edge [6]. Stabilization of protrusion and promotion of migration on substratum is mediated through the transmembrane integrin receptors that bridge the extracellular matrix (ECM) and intracellular actin filaments via adaptive proteins. Integrins are concentrated on the adhesion complexes, especially at the leading and trailing edges, and serve as traction sites for migrating cell adhesion on the substratum [7]. Adhesion assembly and disassembly are constantly and rapidly switched on a migrating cell [5], which is regulated by both protein kinases and phosphatases [8]. FAK is a key tyrosine kinase in linking integrins to downstream Rac-specific GEFs that in turn regulate actin polymerization or turnover [5].

On the other hand, another cytoskeletal component, microtubule, usually provides guidance for cell movement, determines protrusion of the leading edge, and the direction of migration [9–11]. Cdc42, as a master regulator of cell polarity, affects localization of the microtubule-organizing center (MTOC) in front of the nucleus towards the leading edge [10,12].

Mertk receptor tyrosine kinase, belonging to the TAM (Tyro3, Axl and Mertk) family of receptor tyrosine kinases, has recently emerged as an important receptor for phagocytic clearance of apoptotic cells or spent cell debris. Mutations in *Mertk* gene are characterized with accumulation of apoptotic debris in spleen and spent out segments of photoreceptors in retina due to defective phagocytosis, which eventually leads to autoimmune disorders and retinal degeneration [13–19].

Mounting evidence indicates that Mertk affects phagocytic uptakes of apoptotic cells by macrophage and the spent or abandoned cell debris by non-professional phagocytes through activation of its downstream signal transduction pathways. Activation of Mertk has been shown to cause tyrosine phosphorylation of Vav1 that in turn stimulates GDP replacement by GTP on Rac1, Cdc42, and RhoA, [20], and the phosphorylation of FAK and phospholipase Cγ2 in a few cell lines or peritoneal macrophages [21–23]. Mertk may also modulate cytoskeletal rearrangement through activation of PI3K signaling pathways, as shown in a variety of cell types including cancer cells [24], hematopoietic cell [25] and phagocytosing RPE cells [26]. Mertk can drive redistribution of dynamic protein, myosin II, from F-actin bundles to the sites of phagocytic cup [27]. A large body of evidence shows that Mertk plays an essential role in phagocytosis through activation of its downstream signaling which is critical for cytoskeletal rearrangement during phagocytosis.

In the present study, we further studied the functional role of Mertk on attachment, spreading and cell migration. We found that proteose peptone induced peritoneal macrophages exhibited weak initial attachment and spreading when plated on cell culture dishes or glass coverslips. Mertk mutant cells exhibited poorly polarized body morphology, indistinguishable leading and trailing edges, and abnormal MTOC and microtubule localization; as a consequence, the cells lost their highly mobile characters and directional migration patterns. In conclusion, Mertk deficiency affects not only phagocytosis but also cell morphology and mobility, conceivably through a common signaling mechanism used for phagocytosis.

## Materials and Methods

### Animal

The *Mertk* ^*-/-*^ gene knockout mice have been described previously [13,15]. All animals were housed in a pathogen-free facility and handled according to the regulations of the Institutional Animal Care and Use Committee (IACUC) at the University of Louisville. The IACUC and ethics committee at University of Louisville specifically approved this study, with IACUC No.10131.

### Isolation and culture of peritoneal macrophages

Peritoneal macrophages were collected from mature WT and *Mertk* ^*-/-*^ mice at 2–4 months of age, after daily intraperitoneal injection of 200 μL/10g body weight of 10% proteose peptone for 3 days. Peritoneal extrude cells were collected into complete RPMI-1640 medium supplemented with 5% FBS, 1x penicillin/streptomycin, 1x glutamax (Invitrogen, CA). For environmental scanning electronic microscopy (ESEM) and immunofluorescence microscopy, 2×10^5^ cells were plated into each of a 24-well plate containing one glass coverslip per well and cultured at 37°C, 5% CO_2_ for 2 hr. Unattached cells were washed off with RPMI-1640, and the remaining cells were further cultured in complete RPMI-1640 medium for 2 more hr before further process for assays. For cell migration video microscopy, peritoneal extrude was plated onto 35-mm confocal dishes and unattached cells were washed off after 2 hr. The remaining attached cells were further cultured in complete RPMI-1640 medium at 37°C overnight.

### Immunocytochemistry and Western Blotting

The macrophage samples on the coverslip were rinsed once with 1xPBS and fixed with 4% paraformaldehyde for 10 min at RT. After permeabilized with 0.5% Triton X-100/1xPBS for 10 min on ice, cells were blocked in 1xPBS supplemented with 3% normal donkey serum, 0.5% BSA, and 0.5% Tween-20 for 1 hr, and then incubated at 4°C overnight with mouse monoclonal antibodies for Myosin II or alpha tubulin (both were used at 1:200 dilution, Abcam, MA). In some assays, Phalloidin-TRITC (Sigma-Aldrich, USA) was included. After three washes in 1xPBS, the cells were labeled with Alexa Fluor488- conjugated secondary antibody (Invitrogen, USA; 1:200) for 2 hr at RT. The DAPI (Roche Diagnostics, Germany) was used for nuclear staining. Microscopic images were captured and analyzed on a confocal laser-scanning microscope (Leica, Germany).

For Western blotting, 4x10^6^ peritoneal extrude cells were plated onto 100-mm dishes. After 2 hr of initial attachment in complete macrophage culture medium, and then intensive washes with 1xPBS three times, the remaining cells were further cultured at 37°C, 5% CO_2_. In the following day, the thymocytes harvested from 2–4 month old C57BL/6 mice were treated with 2 μM dexamethasone (Sigma-Aldrich, USA) for 12 hr. The treated thymocytes were washed for 3x with 1xPBS before added to macrophage culture with a ratio of 3:1, for 0, 10, 20 and 40 min. After 3x washes to remove unphagocytosed cells, the remaining macrophages were collected in ice-cold 1xPBS, and centrifuged. The cell pellets were directly lysed in RIPA buffer containing 1x complete protease inhibitor cocktail (Roche Diagnostics, Germany). The protein concentration was determined by Bradford method and an equal amount (20 μg) of protein lysates was boiled in 1x gel-loading buffer for 5min and then loaded onto 10% SDS-PAGE gel. After electrophoresis, the proteins were transferred to PVDF membrane. For immunoblotting, the primary antibodies used were rabbit anti-phospho-FAK-Tyro397 (Cell Signaling Technology, Danvers, MA) and mouse monoclonal anti-GAPDH (Sigma-Aldrich, St. Louis, MO). The horseradish peroxidase-conjugated goat anti-mouse or anti-rabbit secondary antibodies and an enhanced chemiluminescence (ECL) system (Amersham Pharmacia, Piscataway, NJ) were used to visualize the signals.

### Environmental Scanning Electron microscopy

The cells grown on glass coverslips were fixed with 2.5% glutaraldehyde in 0.1M sodium cacodylate buffer and stored at 4°C overnight. The samples were washed 3x with 1x PBS, followed by dehydration in an ethanol gradient of 1x30%, 1x50%, 1x70%, 1x85%, 1x95% and 2x100% for 10 min at each step. After dried in a CPD300 automatic critical point dryer (Leica Microsystems, Germany), cells on the coverslips were viewed and images were captured on a Quanta 200 FEG environmental scanning electron microscope (FEI, USA) under high vacuum mode. 180 cells from nine to ten view fields for each sample were selected for measurement of cell body area and perimeter length using the software, Image-Pro Plus 6.0 (Media Cybernetics, USA).

### Video Microscopy

A motorized inverted microscope (model IX81, Olympus, Japan) equipped with differential interference contract (DIC) optics was used for motility assay. Cells cultured in a 35-mm confocal dish with 14-mm hole size (WHB, Inc, Beijing, China) were placed in an observation chamber supplied with 5% CO_2_ and maintained at 37°C. Images were acquired at one frame per 30 seconds for 30mins with a cooled digital imaging camera (MCA-2000U, United Biotechnology, USA) driven by a MCA-2000U Image-pro Science Computer and processed with Andor iQ 1.10.3 (Andor Bio-Imaging Division, United Kingdom) and Adobe Photoshop 5.0.2 (Adobe Systems, CA).

### Statistical analysis

Data were analyzed by two-way analysis of variance ANOVA using ProSTAT ver5.5 for a comparison of the significance of differences between two groups. Data was expressed as mean±SD, and *p* values ≤ 0.05 were considered statistically significant.

## Results

### *Mertk* ^*-/-*^ macrophages show slow attachment and spreading after plating

**Figure 1:**
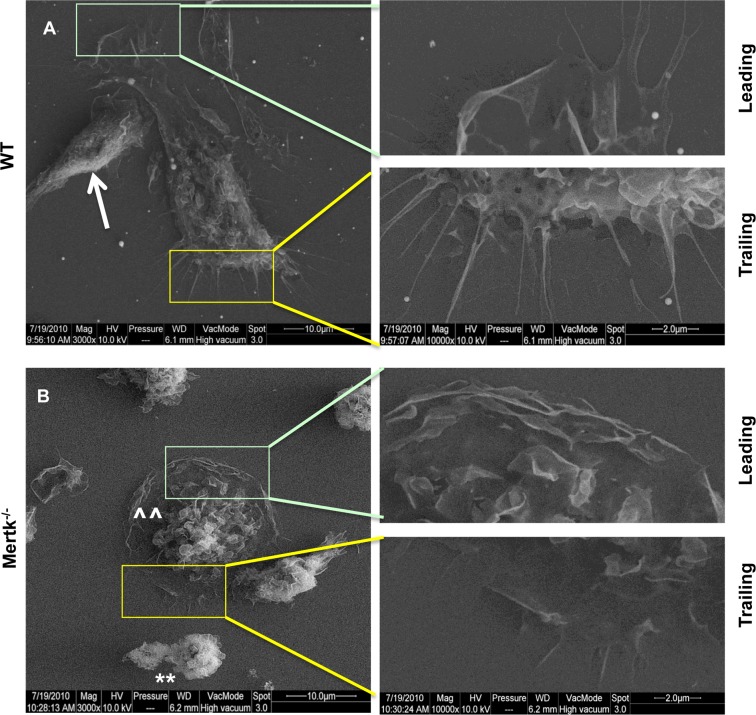
*Mertk* ^*-/-*^ macrophages lose bipolar spindle-like body shape and exhibit no clear leading or trailing edges. (A, B) Proteose Peptone induced peritoneal macrophages were prepared from the WT (A) and *Mertk* ^*-/-*^ (B) mice. The extruded cells were cultured on glass coverslips for 2 hr, followed by wash with 1xPBS to remove unattached cells. After 2 more hr culture, cells were fixed, dehydrated and dried following the procedures described in the Materials and Methods. The cell images were captured by environmental scanning electron microscopy (ESEM), with magnification of 3,000 fold. Two inserts representing the leading and trailing edges from each image were shown on the right with magnification of 10,000 fold.

Mertk affects phagocytosis through the regulation of cytoskeletal rearrangement, which may also be critical for cell attachment and spreading, we therefore examined the effect of the loss of Mertk on the cell attachment on the culture dishes. We prepared peritoneal macrophages after a 3-day proteose peptone induction and plated onto glass coverslips. After 2 hr of recovery from plating and PBS wash, cells were subjected to environmental scanning electron microscopy (ESEM). While WT macrophages started to form spindle-form of morphology (Fig. 1A, arrow), the majority of *Mertk* ^*-/-*^ macrophages barely developed a similar spindle-like cell shape and simply settled on the dish as shown by round cell morphology (Fig. 1B, **). Some of *Mertk* ^*-/-*^ cells were flattened into a round pancake-like shape (Fig. 1B, ^^). The WT cells showed polarized morphology with clear leading and trailing edges, representing the moving head and trailing pole, respectively (Fig. 1A, inserts on the right panel). The leading edges developed into lamellipodium protrusions and shaped with “ruffle” movement; while the trailing edges were retracted to form radical filopodia, indicating that a strong force was created during fast forward migration. Such clear leading and trailing edges on both poles of a moving cell indicated a clear straight forward moving direction. The moving WT cells developed many ruffles at the top of the cells, many of which displayed barbed shaped membrane folding. The cell body or nucleus was located either near the leading edge or on the trailing side depending on the stages of the movement or due to rapid changes in moving directions.

However, such polarized shape was not clearly evident on the *Mertk* ^*-/-*^ macrophages. Because of their slow attachment and spreading, most of the cells remained in a round shape; in some flatten cells, the lamellipodium-like structures developed surrounding the periphery of the cell body. The majority of *Mertk* ^*-/-*^ cells showed unpolarized morphology, and were scarcely accompanied by the spindle-shaped cells. Even in such spindle-shaped cells, there were little filopodia on both poles. It was also difficult to distinguish the moving direction. In addition, the lamellipodium attachment area on the *Mertk* ^*-/-*^ cells was smaller than those seen on the WT cells, and the ruffles were mainly located on the periphery and top of the cells, suggesting a lack of moving force generated from underlying cytoskeletons.

### *Mertk* ^*-/-*^ macrophages show reduced cell area

**Figure 2:**
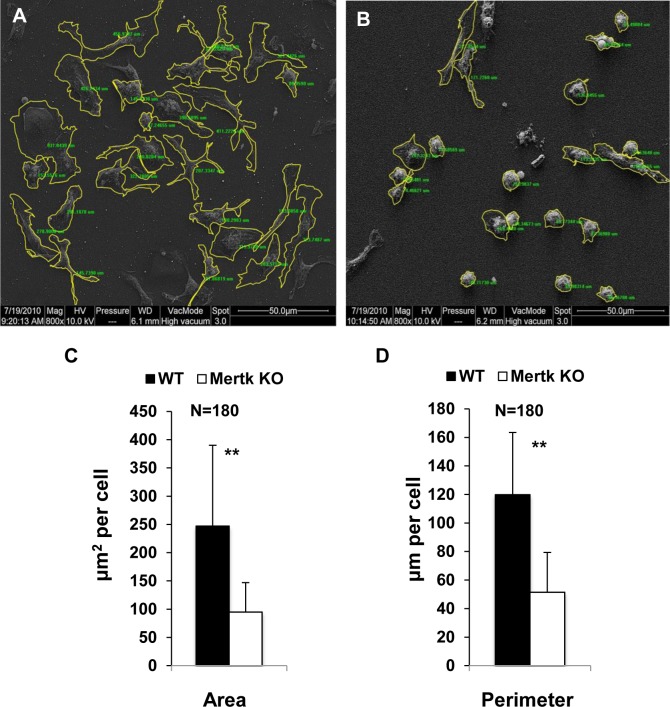
*Mertk* ^*-/-*^ macrophages display decreased cell body area and perimeter length. (A, B) Peritoneal macrophages from the WT (A) and *Mertk* ^*-/-*^ (B) mice were prepared for ESEM microscopy as described in the Fig. 1 legend. The cell boundaries were selected and drawn using ImagePro Plus 6.0. Magnification, 800x. (C, D) The cell body area and perimeter were identified and measured using Image-Pro Plus 6.0 on the ESEM captured images. 180 individual cells were randomly selected from 9–10 review fields for each genotype. Data are expressed as Mean±SD, n = 180. ** indicates p<0.01.

Given that most of the *Mertk* ^*-/-*^ macrophages showed round shape and delayed spreading, we then measured the cell body area and periphery length with ESEM. The proteose peptone induced WT and *Mertk* ^*-/-*^ macrophages were prepared and processed for ESEM observation. The images were captured with a cooled digital imaging camera and the cell body area and peripheral length were analyzed and calculated using Image-Pro Plus 6.0. As shown in Fig. 2, both WT and *Mertk* ^*-/-*^ cells showed significant differences in body area and peripheral length (Fig. 2, A versus B). We selected 180 cells from each sample and measured for the cell area and perimeter using Image-Pro Plus 6.0. The average area for *Mertk* ^*-/-*^ macrophages was approximately 100 μm^2^ in contrast to the average of 250 μm^2^ for the WT cells (Fig. 2C). Similarly, the peripheral length per *Mertk* ^*-/-*^ macrophage was around 55 μm versus the length of 120 μm per WT cell (Fig. 2D). Both parameters showed significant differences (P<0.01) between two groups. These results indicate that Mertk affect cell spreading after attachment on the culture substance.

### *Mertk* ^*-/-*^ macrophages lose polarized distribution of cytoskeletal filaments and dynamic motor protein

**Figure 3:**
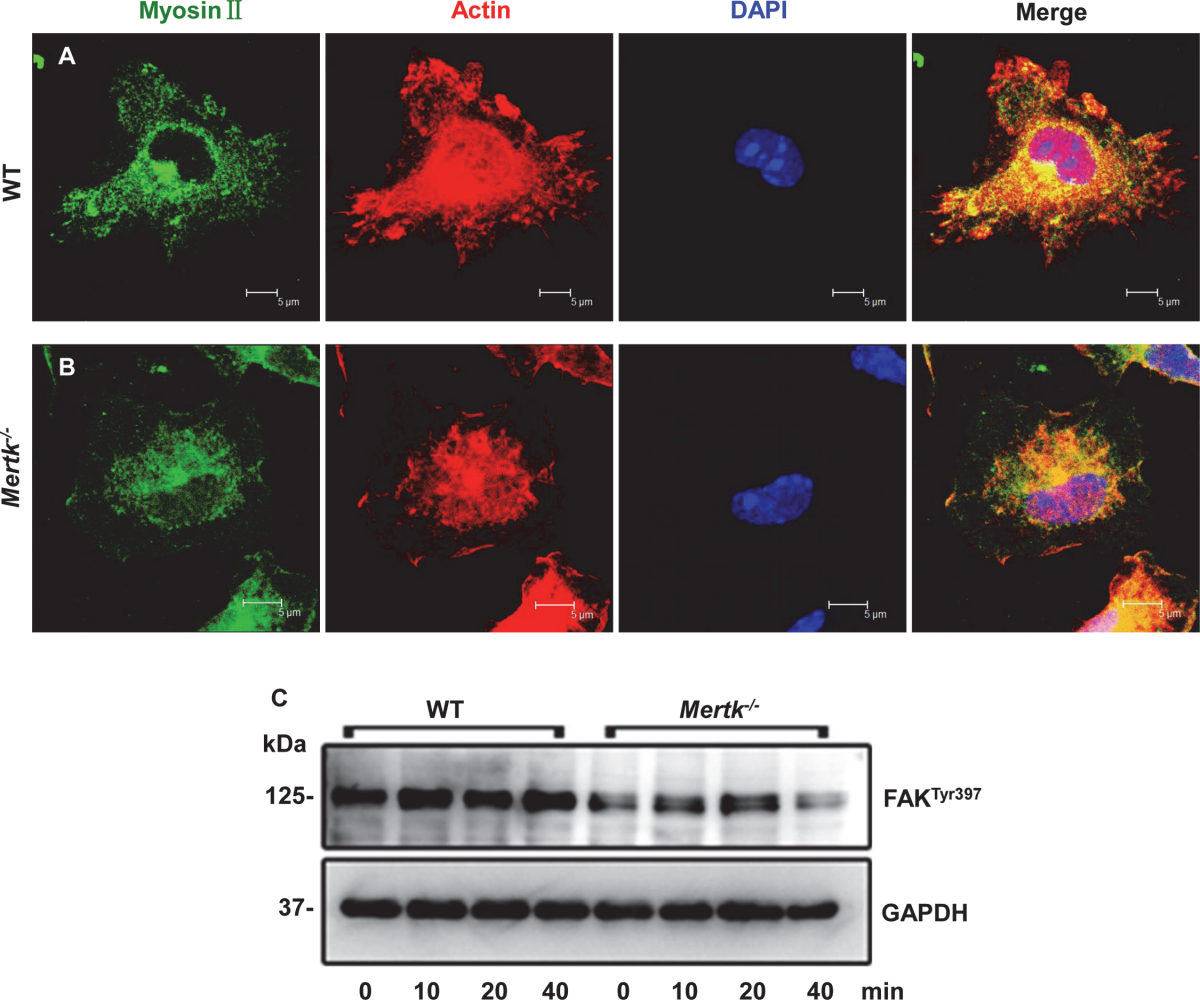
*Mertk* ^*-/-*^ macrophages exhibit unpolarized distribution of filamentous actin and myosin II, and decreased FAK phosphorylation. Peritoneal macrophages from the WT (A) and *Mertk* ^*-/-*^ (B) mice were prepared following the procedure described in the Materials and Methods. After fixture in 4% paraformaldehyde for 10 min at RT, cells were blocked in 1x PBS containing 3% normal donkey serum, 0.5% BSA and 0.5% Tween-20, for 1 hr, then incubated with mouse monoclonal antibodies against Myosin II (1:200 dilution, Abcam, MA) and TRITC-conjugated phalloidin (Sigma-Aldrich, MO) in the blocking buffer at +4°C overnight. After washed for 3x with 1xPBS plus 0.5% Tween-20, the cells were incubated in the Alexa Fluor 488-conjugated donkey anti-mouse secondary antibody at RT for 1 hr. Myosin II and F-actin were labeled green and red, respectively. Nuclei were counterstained blue with DAPI (Roche Diagnostics, Germany). Microscopic images were captured and analyzed on a confocal laser-scanning microscope (Leica, Germany). Scale bars in (A) and (B), 5 μm. (C) Western blotting shows decreased FAK phosphorylation at tyrosine 397 after addition of apoptotic T cells for the indicated time points. GAPDH was used as control for indication of equal protein loading.

Cell migration on supporting substance relies on rapid actin polymerization on the leading edge [2–4]. To characterize whether Mertk mutation affected cytoskeletal arrangement and therefore cell spreading and movement, we stained cells with TRITC-labeled phalloidin that selectively stained F-actin filaments, and found that actin filaments unevenly distributed on the leading edges in addition to the cell body in the WT cells (Fig. 3A). This was also true for myosin II that was found on the cell edges (Fig. 3A). However, neither F-actin nor myosin II was polarly distributed around the periphery of the *Mertk* ^*-/-*^ macrophage (Fig. 3B). Instead, they were mainly concentrated in the cell body. This result suggests that Mertk regulates actin polymerization and dynamic protein myosin II recruitment on the leading edge of cells.

Since cell migration depends on cytoskeletal rearrangement and rapid adhesion disassembly and assembly involving integrins and their downstream FAK, we then examined the FAK phosphorylation on the *Mertk* ^*-/-*^ macrophages simulated with apoptotic cells for period times. Phosphorylation at FAK-Tyr397 was decreased and apoptotic encountering did not increase the phosphorylation signals, as compared with the results of the WT cells (Fig. 3C). This suggests that Mertk affects FAK phosphorylation in macrophages.

### Mertk regulates cell directional movement

**Figure 4:**
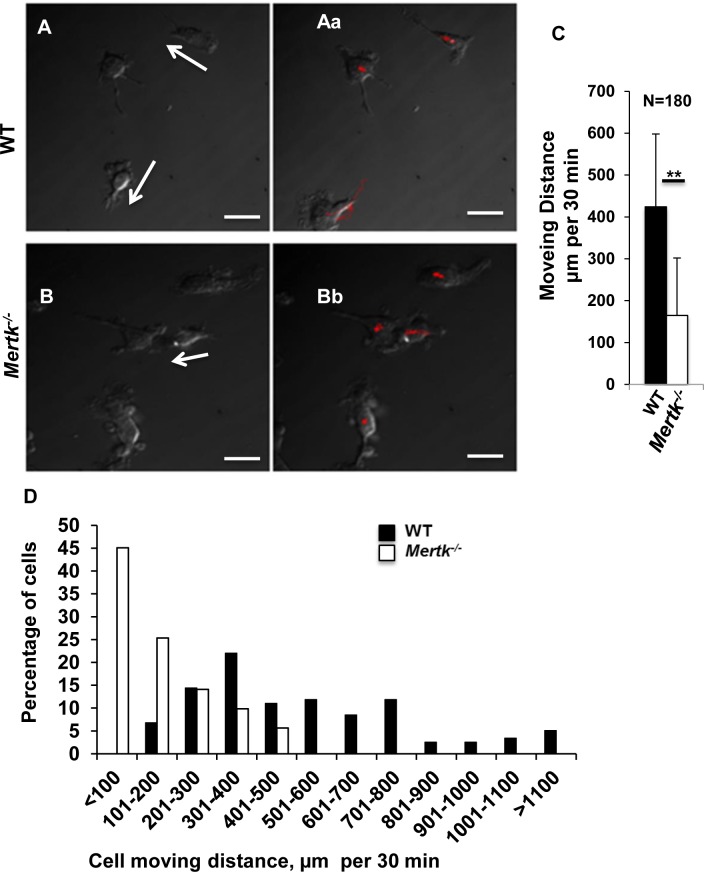
*Mertk* ^*-/-*^ macrophages display no directional movement. Peritoneal macrophages from the WT (A) and *Mertk* ^*-/-*^ (B) mice were prepared following the procedure described in the Materials and Methods, and cultured in a 35-mm confocal dish inside a chamber supplied with 5% CO_2_ and a controlled temperature of 37°C on an inverse microscope. Images were acquired at one frame per 30 seconds for 30mins with a cooled digital imaging camera (MCA-2000U, United Biotechnology, USA) and analyzed by Andor iQ 1.10.3 (Andor BioImaging, UK). (C) The moving distance per cell within 30 min was monitored and measured from 180 individual cells in each genotype. Data are presented as Mean±SD, n = 180, ** indicates p value < 0.01. (D) Data are shown as the percentage of cells that moved at an indicated distance in μm per 30 min. The majority of the *Mertk* ^*-/-*^ macrophages moved less than 200 μm per 30 min, and cells moving more than 501 μm per 30 min were rarely found whereas nearly a quarter of al WT cells moved 301–400 μm within 30 min and approximately half of the WT cells moved at distance more than 501 μm per 30 min. Scale Bars in (A) and (B), 10 μm.

Given that Mertk affects actin polymerization and dynamic protein distribution on the leading edge of the moving cells, we then measured whether Mertk mutation affected cell movement. Under DIC microscope, we observed rapid directional movement on the WT cells, and such movement was accompanied with sudden changes in moving direction, and sometimes the exchange of position in the leading and trailing edges, i.e., the leading edge became the trailing edge and vice versa (Fig. 4A). However, the *Mertk* ^*-/-*^ macrophages lost such directional movement, and mainly conducted oscillating movement around the cell body (Fig. 4B). It was obvious that the WT cells moved faster and at a longer distance than did the *Mertk* ^*-/-*^ cells. We next traced the moving distance for 180 individual cells. Each of them was recorded for 30 minutes and it was found that the average moving distance for the WT cells were more than 500 μm whereas, under same experimental conditions, the *Mertk* ^*-/-*^ cells moved less than 200 μm on average, with significant reduction in moving distance per cell at any given time period (Fig. 4C). This experiment was repeated thrice with similar conclusion. In all of these experiments, we found that more WT cells moved with longer distance and more *Mertk* ^*-/-*^ cells moved at a shorter distance (Fig. 4D). The data indicates that Mertk affects cell movement, presumably via regulation of cytoskeletal rearrangement.

### Mertk deficiency alters microtubule organization and microtubule-organizing centers (MTOCs) position

**Figure 5:**
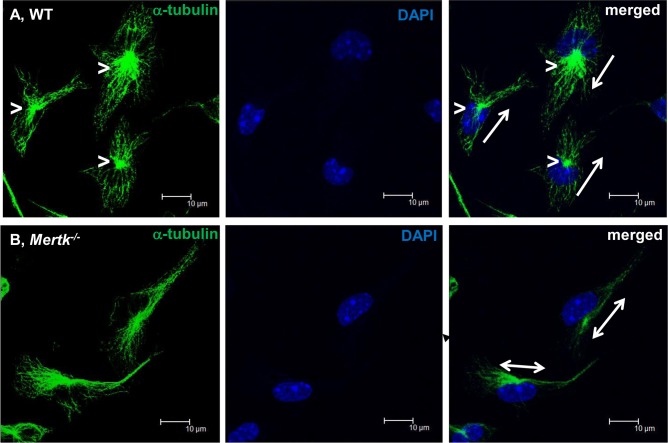
*Mertk* ^*-/-*^ macrophages show disorganized microtubule distribution and undetectable MTOC. The WT (A) and *Mertk* ^*-/-*^ (B) macrophages were immunostained using mouse monoclonal anti-α-tubulin antibody (left panel), and the nuclei were stained blue by DAPI (middle). The merged images are shown on the right panel. The immunostaining procedure is described above. Arrows in (A) or double headed arrows in (B) indicate the cell moving directions, and the symbol “>” points to MTOC. Images were captured with a Leica confocal microscope equipped with a cooled digital imaging camera. Scale bar, 10 μm.

Actin filaments and microtubules and their associated proteins play an important role in the generation of driving force and in the determination of the direction of migration. It is well documented that disruption of microtubules inhibits directional migration but not random movement [28–30]. As explained above, *Mertk* ^*-/-*^macrophages lost their directional migration and instead exhibited random oscillating movement. We then asked whether microtubules and MTOC were disorganized in the mutant cells. The microtubules were immunostained using anti alpha-tubulin specific antibody and observed on a confocal microscope. A comparison of the two genotypes of macrophages revealed a striking difference in microtubule organization, and MTOC structure and location. MTOCs were clearly evident and orientated in the front of moving WT nuclei, from which the radical microtubules emanated (Fig. 5A). On the other hand, the microtubule bundles in the mutant cells were mainly located on one side of cell body, and the MTOC was not readily developed (Fig. 5B). This result shows that loss of Mertk affects MTOC development and microtubule organization, which may be responsible for the loss of directional migration seen on *Mertk* ^*-/-*^mutant macrophages.

## Discussion

Cell migrates directionally in response to developmental clues or chemoattractants. Cell surface receptors, including GTP-binding protein (G protein)-coupled receptors and receptor-type tyrosine kinases play important roles in the transduction of extracellular clues into intracellular signaling molecules. It triggers the recruitment and activation of downstream master regulators, well studied of which are Rac, Cdc42 and RhoA, for actin polymerization and new microtubule network formation.

Mertk has been demonstrated to be a critical receptor tyrosine kinase in regulation of cytoskeletal rearrangement and phagocytosis [13–19]. Mertk participates in regulation of phagosome ingestion, possibly by activating its downstream signaling cascades. The current model for signal transduction in regulation of phagocytosis is based on recent discoveries [21,22,31–33]. Binding of rod OS to αvβ5 integrin on retinal pigmental epithelium (RPE) results in phosphorylation of focal adhesion kinase (FAK) at tyrosine-397, which is essential for Mertk-mediated particle engulfment and integrin signaling in cytoskeletal changes [32,34]. In addition to FAK, other src homology 2 (SH2) domain-containing proteins can also interact with Mertk. The guanine nucleotide exchange factor Vav1 is associated with a chimeric Mertk in 293T cells before activation, and was released upon receptor activation [20]. Phospholipase-Cγ2 was phosphorylated and associated with Mertk in peritoneal macrophages upon Mertk crosslinking [23]. Significantly, Mertk-mediated engulfment of apoptotic cells led to the formation of the GTPase complex CrkII/Dock180/Elmo, a conserved set of adaptor proteins which is associated with Rac1and Cdc42 GTPase activation to control changes in the actin cytoskeleton during uptake [21].

While Mertk in phagocytosis have been intensely studied, its functional role in cell migration has not been readily investigated. In the present study, the macrophages lacking Mertk receptor exhibited unpolarized morphology *in vitro*, peri-nucleus localization of actin filaments and myosin II dynamic protein. The mutant macrophages lost their directional movement, which was replaced with mainly the oscillating swinging around the cell body. The average directional migration distance in any given time frame was dramatically decreased. Our observation on the *Mertk* ^*-/-*^ macrophage with altered morphology and impeded mobility is consistent with what seen in the Mertk-inhibited glioblastoma cells in which the Mertk expression was knocked down with shRNA [35]. Those Mertk-inhibited glioblastoma cells exhibited slender cell bodies with focal granulations, increased number of focal adhesion, diffuse and disorganized stress fiber. Western blotting revealed an increased phosphorylation of FAK at Tyr397 and upregulated expression of RhoA GTPase, which was postulated to be causative for decreased cell migration and altered cellular morphological changes [35]. However, although we observed the similar abrogated morphological and migrating phenotypes on the Mertk mutant macrophages, we conversely obtained a decreased phosphorylation of FAK at Tyr397 in the mutant cells as compared to this of the WT counterpart upon induction with the apoptotic cells. Phosphorylation of FAK at Tyr397 is critical for cell morphology and migration through interaction with several downstream SH2-adaptor proteins, which affects dynamic assembly or disassembly of focal adhesion, establishment of a proper leading edge and maintenance of the polarity of moving cells [36–40]. Conditional knockout of FAK in macrophages caused marked inability to form stable lamellipodia necessary for directional locomotion [41]. Fibroblasts with FAK null mutation or expressing a Tyr397-mutated FAK protein showed a rounded morphology and defective cell migration [34]. On the other hand, a constitutively active form of Mertk expressed in the 293T cells caused phosphorylation and recruitment of FAK to αvβ5 integrin, and induced p130cas/CrkII/Dock180 formation and Rac1 activation [21,22]. It is well documented that FAK affects cytoskeletal reorganization through recruitment of the p130^cas^/Crk/Dock180/ELMO complex that regulates Rac1 [21,31,32,42]. It is conceivable that impaired phosphorylation on FAK at Tyr397 observed in the present study is likely responsible for the altered cell morphology and loss of the directional mobility seen in the *Mertk* ^*-/-*^ cells.

Furthermore, in comparison with WT cells, the Mertk deficient macrophages did not exhibit a clear MTOC and also lacked MTOC-associated, radial-distributed bundles of microtubule filaments, which were clearly shown in the WT cells. MTOC is considered as an organizer capable of initiating microtubule polymerization from tubulins. The MTOC position and its associated microtubule network are important in the development of lamellipodia and establishment of the directional migration [43,44]. In the *Mertk* ^*-/-*^ cells, microtubule bundles were mainly localized on one side of cell body and extended toward both sides relative to the nuclei. It is well established that microtubules are important for directional movement and disruption of the microtubule formation inhibits cell directional migration but not for random movement [28–30]. Such random movement was observed on the *Mertk* ^*-/-*^ cells. Our results indicate that Mertk mutation not only affects the polymerization of actin filaments and the distribution of dynamic protein myosin II, but also MTOC formation and localization. Collectively, this abolished the directional movement by *Mertk* ^*-/-*^ macrophages.
